# Automated Photochemically Induced Method for the Quantitation of the Neonicotinoid Thiacloprid in Lettuce

**DOI:** 10.3390/molecules24224089

**Published:** 2019-11-12

**Authors:** J. Jiménez-López, E.J. Llorent-Martínez, S. Martínez-Soliño, A. Ruiz-Medina

**Affiliations:** Department of Physical and Analytical Chemistry, Faculty of Experimental Sciences, University of Jaén, Campus Las Lagunillas, E-23071 Jaén, Spain; jujimene@ujaen.es (J.J.-L.); saramsolinho@gmail.com (S.M.-S.)

**Keywords:** neonicotinoid, thiacloprid, solid-phase spectroscopy, optosensor, luminescence

## Abstract

In this work, we present an automated luminescence sensor for the quantitation of the insecticide thiacloprid, one of the main neonicotinoids, in lettuce samples. A simple and automated manifold was constructed, using multicommutated solenoid valves to handle all solutions. The analyte was online irradiated with UV light to produce a highly fluorescent photoproduct (λ_exc_/λ_em_ = 305/370 nm/nm) that was then retained on a solid support placed in the flow cell. In this way, the pre-concentration of the photoproduct was achieved in the detection area, increasing the sensitivity of the analytical method. A method-detection limit of 0.24 mg kg^−1^ was achieved in real samples, fulfilling the Maximum Residue Limit (MRL) of The European Union for thiacloprid in lettuce (1 mg kg^−1^). A sample throughput of eight samples per hour was obtained. Recovery experiments were carried out at values close to the MRL, obtaining recovery yields close to 100% and relative standard deviations lower than 5%. Hence, this method would be suitable for routine analyses in quality control, as an alternative to other existing methods.

## 1. Introduction

Neonicotinoid pesticides are the most widely used class of insecticides worldwide, representing a 25% share of the insecticides market in 2014 [1]. They have a wide range of applications: plant protection (crops, vegetables, and fruits), veterinary products, and biocides to invertebrate pest control in fish farming. However, their use is a controversial subject, as several toxicological studies proved that some neonicotinoids (imidacloprid, clothianidin, and thiamethoxam) produce the collapse of honey-producing bee colonies [2]. In 2018, the European Union decided to ban the outdoor use of these three pesticides [3], and the Environmental Protection Agency announced on May 2019 that the registration for 12 neonicotinoid-based products would be canceled. However, the mentioned ban does not affect thiacloprid (TCP) and other neonicotinoids, which makes it important to develop accurate and quick analytical methods for their reliable quantitation in a wide variety of food samples, in order to ensure their safe consumption.

Among neonicotinoids, TCP is one of the most commonly used, and it belongs to the so-called “first generation” neonicotinoids. The usual analytical methods for TCP quantitation in food samples use liquid chromatography [4,5,6,7,8]. In particular, HPLC-MS/MS [9,10] and UHPLC-MS/MS [11,12] have been reported for their determination in lettuce. Moreover, electrochemistry [13,14], micellar electrokinetic chromatography [15], immunoassays [16,17], and luminescence [18,19,20] have been also proposed for TCP quantitation. The main goal of this work was to develop an alternative luminescence analytical method for TCP routine analysis in lettuce, one of the most widely consumed vegetables, paying special attention to the simplicity, economy, and sample throughput of the system developed.

The use of luminescence sensors has increased in the last decade, minimizing reagents consumption and increasing the degree of automation. In this sense, the use of automated methodologies, such as multicommutated devices, provide advantages, such as increased precision, robustness, and high automation. The combination of flow methodologies and solid-phase spectroscopy (SPS) is a successful approach that maintains the key advantages of automated flow systems, increasing the sensitivity and selectivity of the analytical methods due to the retention and pre-concentration of the target compounds on a solid support placed in the detection area [21]. For instance, a previous method was reported that used sequential injection analysis for the fluorometric determination of hydroxytyrosol (phenolic phytochemical with antioxidant properties in vitro) in food samples, measuring its native fluorescence [22]. The use of multicommutation has proved successful, too, for the quantitation of clothianidin by photochemically induced fluorescence (PIF) in drinking water, rice, and honey [23]. As a follow-up to previous works, we report a multicommutated flow-injection analysis (MCFIA)-based method, using PIF detection to overcome the handicap of the absence of native fluorescence of TCP. The main difference from the previous paper is the selected food sample, lettuce, which made it necessary to carry out a different extraction procedure due to the different matrix. In addition, the novel instrumental and chemical conditions made it possible to discriminate between TCP and other neonicotinoids. The analyte is UV-irradiated to produce a fluorescence photoproduct which is retained and detected on a solid support placed in the flow cell. By means of the MCFIA manifold, this irradiation takes place online, simplifying the procedure and increasing sample throughput. The proposed method allows for the fulfillment of the Maximum Residue Limit (MRL) of the European Union [24] for TCP in lettuce.

## 2. Experimental

### 2.1. Reagents and Solutions

TCP (Sigma-Aldrich, Madrid, Spain) stock solution of 100 mg L^−1^ was prepared in Milli-Q water (Millipore); it was kept in the dark at 4 °C, and working solutions were prepared daily. Acetonitrile, graphitized carbon black (GBC), primary–secondary amine (PSA), hydrochloric acid (HCl), sodium hydroxide (NaOH), sodium acetate, acetic acid, ammonium chloride (NH_4_Cl), ammonia (NH_3_), and magnesium sulphate (MgSO_4_) were purchased from Sigma (Sigma-Aldrich). Isolute QuEChERS extraction kit was acquired from Biotage (Sweden). Sephadex QAE A-25 and Sephadex SP C-25 in sodium form, both of them 40–120 μm average particle size (Sigma-Aldrich, Buchs, Switzerland), and C_18_ bonded phase silica gel beads (Waters, Milford, MA, USA) of 55–105 μm average particle size, were tested as solid supports.

Ultrapure water (Milli-Q Waters purification system, Millipore, Milford, MA, USA) was used for all analyses.

### 2.2. Instrumentation and Apparatus

A Cary-Eclipse Luminescence Spectrometer (Varian Inc., Mulgrave, Australia) with Cary-Eclipse (Varian) software and a Hellma flow cell 176.752-QS (Hellma, Mülheim, Germany) (25 µL of inner volume, and a light path length of 1.5 mm) were used. The cell was filled with the solid support and was blocked at the outlet with glass wool, to prevent displacement of the particles.

A four-channel Gilson Minipuls-3 (Villiers Le Bel, France) peristaltic pump with rate selector and methanol-resistant pump tubes type Solvflex (Elkay Products, Shrewsbury, MA, USA) were used. An electronic interface based on ULN 2803 integrated circuit (Motorola, Phoenix, AZ, USA) was employed to generate the electric potential (12 V) and current (100 mA) required to control the three 161T031 NResearch three-way solenoid valves (Neptune Research, West Caldwell, NJ, USA). The software for controlling the system was written in Java. Flow lines of 0.8 mm internal diameter PTFE tubing and methacrylate connections were used.

For UV-irradiation, a homemade continuous photochemical reactor was constructed by coiling PTFE tubing (180 cm, 0.8 mm i.d.) around a low-pressure mercury lamp (30 W, 254 nm). A Sonorex Digital 10P (Bandelin Electronic, Berlin, Germany) ultrasonic bath, a pH-meter Crison GLP21 (Crison Instruments, Barcelona, Spain), a centrifuge Mixtasel-BL (Selecta, Barcelona, Spain), and a rotary evaporator (Heidolf, Schawabach, Germany) were also used.

### 2.3. Sample Preparation

All samples (iceberg lettuce, baby Romaine lettuce, and green oak leaf lettuce) were purchased at local markets. Approximately 200 g of each sample was ground and homogenized with a high-speed laboratory homogenizer. TCP was extracted, following a modified QuEChERS method [25]. An extraction kit (Isolute QuEChERS) containing GCB was used for all samples. This nonpolar sorbent allowed for the removal of hydrophobic interaction-based compounds, such as chlorophyll and carotenoids. The method used was as follows: 10 g of sample was weighed in a 50 mL PTFE centrifuge tube, and acetonitrile (10 mL) was added. Then, the content of a 15 mL tube extraction kit (4 g of MgSO_4_, 1 g of sodium citrate, 0.5 g of sodium citrate sesquihydrate, and 1 g of NaCl) was added, and the samples were vortexed for 1 min. After centrifugation (5 min, 4000 rpm), 6 mL of the supernatant was transferred into a 15 mL dispersive SPE tube containing 150 mg of PSA, 900 mg of MgSO_4_, and 15 mg of GCB. Samples were vortexed for 1 min and centrifuged for 5 min, at 4000 rpm. In this way, the acetonitrile (supernatant) contained the analyte. Prior to analysis, an appropriate volume of the acetonitrile extract was diluted with acetate buffer (0.05 mol L^−1^, pH 4.6).

### 2.4. General Procedure

**Figure 1:**
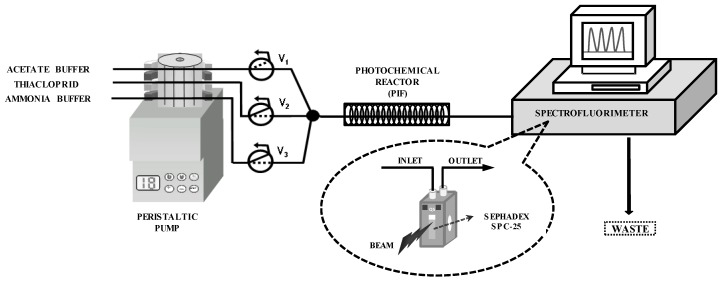
Flow manifold. Vi = three-way solenoid valves.

The flow manifold is shown in Figure 1. In the initial status, all valves are switched off, and the carrier (0.05 mol L^−1^ acetate buffer, pH 4.6) flows through the flow-through cell, while all other solutions are recycled to their vessels. The sample (20–250 µg L^−1^ prepared in 0.05 mol L^−1^ of acetate buffer, pH 4.6) is introduced by simultaneously switching valves V_1_ and V_2_ on for 200 s. In this way, the analyte is carried toward the photochemical reactor, where it is UV-irradiated for 150 s, obtaining its fluorescent photoproduct. Then, the photoproduct is carried toward the flow cell, which is filled with Sephadex SP C-25 microbeads. TCP photoproduct is strongly retained on the solid microbeads, and the fluorescence signal is recorded (λ_exc_/λ_em_ = 305/370 nm/nm). Then, an eluting solution (0.05 mol L^−1^ of NH_3_/NH_4_Cl buffer, pH 9.0) is inserted into the system by activating valves V_1_ and V_3_ for 50 s, desorbing the photoproduct from the solid support. Finally, the carrier solution flows again through the system until the next sample insertion. All calibration standards and samples were analyzed in triplicate.

## 3. Results and Discussion

Neonicotinoids do not present native fluorescence (or very low luminescence in some cases). Therefore, different strategies are required to develop luminescent analytical methods for their determination. In this case, we tested the possibility of irradiating TCP with a UV-lamp, in order to generate possible fluorescent photoproducts. The absorption spectrum of thiacloprid (200–380 nm; maximum at 242 nm) makes this compound an interesting candidate to perform PIF with the low-pressure mercury lamp (emission of 200–300 nm; maximum at 254 nm). The different parameters of the system were optimized to obtain the highest sensitivity.

### 3.1. Instrumental Variables and Selection of Solid Support

We tested different solid supports (Sephadex QAE A-25, Sephadex SP C-25, and C_18_ silica gel) in the flow cell, to select the optimum one for the retention of TCP photoproduct. The optimum sample pH was obtained for pH values of 4–6 (see Section 3.2); as expected, TCP photoproduct was not retained on the anion-exchange QAE A-25, which is suitable for anionic species at basic pH values. On the other hand, although both the cation-exchange SP C-25 and nonionic C_18_ silica gel beads could retain the photoproduct, the signal obtained in C_18_ was very low, observing the highest signal with SP C-25, which was the selected solid support. However, it is important to consider that, when the signal is recorded on a solid support, there is a considerable background signal. Therefore, instrumental parameters have to be carefully studied to achieve the maximum sensitivity without compromising the linear dynamic range due to a high background signal. Excitation and emission slit widths were optimized between 5 and 20 nm, whereas the voltage of the photomultiplier tube (PMT) was studied in the range of 400–800 V. Wide slit widths and high PMT voltages increased the sensitivity, as well as the background signal produced by the solid support. Overall, the best results were obtained for excitation/emission slit widths of 5/10 nm/nm, respectively, and a PMT voltage of 780 V.

### 3.2. Chemical Variables

The chemical variables can affect the performance of the analytical methods not only from the point of view of the generation of the fluorescent photoproduct but also in terms of its retention/elution kinetics on the solid support. We thus optimized the pH value of the sample solution in the first place, adjusting the pH with HCl and NaOH solutions.

**Figure 2:**
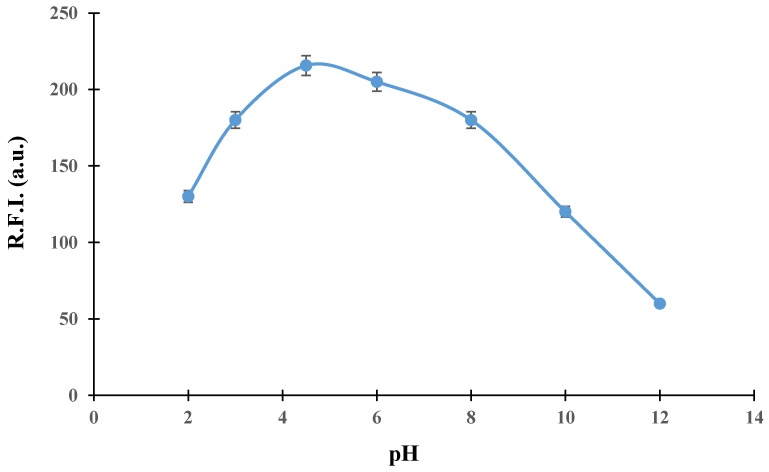
Effect of pH values on the analytical signal.

TCP generated fluorescence photoproducts in a wide range of pH values, obtaining the highest sensitivity in the range of 4–6. For acidic pH values, low luminescence was obtained. Likewise, the luminescence signal decreased drastically as the pH value increased (see Figure 2). Different buffer solutions were tested at this range (acetate, citrate, and succinate), observing an enhancement of approximately 20% when using an acetate buffer solution respect to adjust only with HCl solution. The concentration of acetate buffer was tested in the range 0.01–0.1 mol L^−1^, selecting as optimum 0.05 mol L^−1^ and a pH value of 4.6. However, when using this buffer in both carrier and sample solutions, the photoproduct was not completely eluted from the Sephadex SP C-25 solid support, due to its high retention. An increase in carrier ionic strength (higher buffer concentrations) resulted in a decrease of the analytical signal, as a consequence of a lower retention of TCP photoproduct on the solid support in these conditions. We thus introduced an additional eluting solution to regenerate the solid support after the photoproduct had developed its analytical signal. Due to the nature of the solid support (cation-exchanger), changes in pH values resulted in different retention/elution kinetics of TCP photoproduct. Eluting solutions with pH higher than 8 provided the desorption of the photoproduct, hence selecting a solution of ammonia/chloride ammonium of 0.05 mol L^−1^, at pH 9 (tested in the range 0.01–0.1 mol L^−1^). In this way, TCP photoproduct provided the highest sensitivity when retained on the solid support at pH 4.6, and, after the signal was recorded, the solid support was regenerated by the eluting solution at pH 9.

### 3.3. Irradiation Time

**Figure 3:**
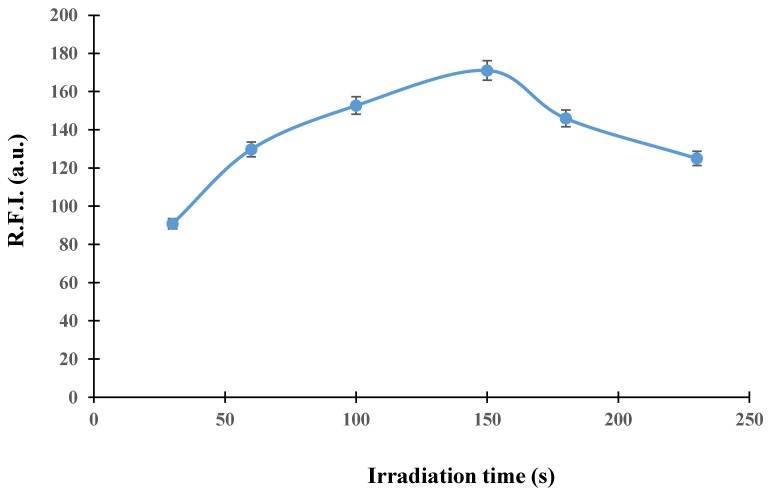
Effect of irradiation time on the analytical signal.

The irradiation time is an essential variable for the generation of fluorescent photoproducts. To optimize this parameter, different UV lamps (8, 15, and 30 W) and irradiation times (30–230 s) were tested for a TCP solution of 100 µg L^−1^. First of all, the 30 W UV lamp was selected, as a higher analytical signal was obtained compared to the other lamps. Second, the irradiation time was studied with this lamp, inserting the sample solution in the system and stopping the flow when the whole plug of the sample was within the photoreactor. Then, the sample was irradiated for increasing periods of time; the results are shown in Figure 3. The analytical signal increased up to an irradiation time of 150 s, decreasing for higher values. The shape of the irradiation time curve suggests a two-step photolysis mechanism, in which the photoproduct observed at 150 s suffered a posterior photodegradation into nonfluorescent product(s) or different photoproduct(s) with lower fluorescence emission. This kind of behavior was previously reported for imidacloprid [26]; hence, the irradiation time was fixed at 150 s. To obtain this irradiation time without the need to stop the flow, flow parameters were optimized. Although the exact structure of TCP photoproduct could not be elucidated, a previous work reported the formula of the photoproduct as C_10_H_11_N_4_OS [27]. This means that TCP (C_10_H_9_ClN_4_S) suffered a C–Cl bond cleavage to produce the photoproduct; the loss of Cl results in an enhancement of the fluorescence.

### 3.4. Flow Parameters

The flow rate selected for the manifold is critical to improving the sample throughput of the analytical method. However, it can also affect it in other ways: (a) a high flow rate may produce overpressures due to the solid support placed in the flow cell; (b) the flow rate and photoreactor length are critical to keeping the optimum irradiation time. As a result, a flow rate of 1.3 mL min^−1^ was selected. Using this flow rate, the length of the photoreactor was adjusted so that the sample plug required 150 s to go through the whole photoreactor.

In flow-through optosensors, the sensitivity of the method improves by increasing the sample volume inserted. The higher the sample volume (keeping the same concentration), the higher the amount of analyte inserted in the system and retained on the solid support. In this way, a pre-concentration of the analyte takes place on the solid microbeads. However, increases in sample volumes also imply lower sample throughput, so a compromise solution is usually needed. When multicommutation is used, sample insertion time, instead of sample volume, is used (when the time and flow rate are known, the volume can be calculated). We thus checked the influence of sample-insertion times between 20 and 300 s. The analytical signal increased up to 200 s, being constant for higher insertion times; hence, 200 s was selected as the optimum insertion time, achieving the required sensitivity for the applications.

### 3.5. Analytical Parameters

The analytical parameters of the system were studied under the optimized conditions previously discussed. They are shown in Table 1, and all of them correspond to a sample insertion time of 200 s.

The calibration graph was constructed, fitting the data by standard least-squares treatment. Detection and quantitation limits, calculated by the 3σ and 10σ criterion, were 6 and 20 µg L^−1^, respectively. Considering sample preparation, these values corresponded to method detection and quantitation limits of 0.24 and 0.8 mg kg^−1^, respectively, in real samples. Precision was studied by analyzing lettuce extracts spiked with 50–200 µg L^−1^ of TCP. Repeatability (*n* = 10, within the same day) and intermediate precision (*n* = 9, 3 consecutive days) were lower than 5% and 8%, respectively. A sample throughput of approximately eight samples per hour was obtained. We also evaluated the robustness of the method by modifying some parameters from the optimum values: excitation/emission wavelengths (±2 nm), photomultiplier tube voltage (±10 V), and flow rate (±0.1 mL min^−1^). The differences observed were always lower than 5% compared to the optimum values. When the proposed method is compared with other methods for TCP determination (Table 2), it can be observed that the precision is similar or better. When comparing the sensitivity, chromatographic methods usually present lower detection limits. However, the detection limit of the present method compares favorably with other non-chromatographic methods.

### 3.6. Interference Study

We studied the potential interference caused by different pesticides that may be present in the analyzed samples (Table 3). Among them were some fluorescent common pesticides (carbendazim and o-phenylphenol), neonicotinoids (clothianidin, imidacloprid, nitenpyram, and thiamethoxam), and other pesticides that have been found in lettuce samples (cypermethrin, chlorpyrifos, and λ-cyhalothrin) [29]. This study was performed by analyzing a solution of 50 µg L^−1^ TCP, adding increasing concentrations of each individual pesticide. Tolerance level was the level of interferent that caused an error of ±3% compared to the analytical signal obtained in the absence of the potential interference. In all cases, interferent levels similar or higher than TCP concentration did not produce any deviation of the analytical signal, therefore allowing the selective quantitation of TCP in the presence of other pesticides that may be present in lettuce samples.

### 3.7. Analytical Applications

The proposed method was applied to the determination of TCP in lettuce samples, in which the MRL established by the European Union is 1 mg kg^–1^ [22]. First of all, extracts of all samples (iceberg lettuce, baby Romaine lettuce, and green oak leaf lettuce) were analyzed to check the absence of TCP (or other potential pesticides that may cause any interference). Neither of the extracts produced an analytical signal. Then, two calibration graphs were prepared for each sample, using external calibration and standard-addition methodology. Some of the samples presented a matrix effect; in this case, standard addition was used for the quantitation of TCP. As none of the extracts had TCP, recovery experiments were carried out to assess the accuracy of the analytical method. Taking into account TCP MRL in lettuce samples, this study was performed by spiking the samples at analyte levels between 0.8 and 10 mg kg^−1^ (spiking was done previously to QuEChERS extraction).

Recovery yields varied between 91% and 108% in all cases (Table 4), with relative standard deviations (RSD; *n* = 3) lower than 5%, confirming the accuracy and precision of the proposed method. We also checked the accuracy by the method of the average recovery [30], obtaining an experimental *t* value of 0.093, lower than the tabulated *t* value at a 95% confidence level.

## 4. Conclusions

MCFIA-SPS-PIF implementation is a very attractive and fruitful research field. In this work, a novel and sensitive optosensor for the quantitation of TCP in vegetables was developed by using QuEChERS for sample pretreatment. The online photodegradation of TCP takes place, followed by the preconcentration and detection of its fluorescent photoproduct on a solid support placed in the flow cell (detection area). The high selectivity obtained is the result of the use of this solid support, as well as the derivatization of the analyte, allowing the fulfillment of the MRL of the European Union for TCP in lettuce.

Chromatographic methods are usually employed for the determination of TCP, but they require many cleanup steps and expensive instruments. However, this study’s results show that flow-through optosensors are a good option for the analysis of neonicotinoids in food. Recoveries close to 100% are obtained in all cases. The simplicity, robustness and high sample frequency of the method developed makes it an interesting prescreening alternative.

## Figures and Tables

**Table 1:** Analytical parameters.

Parameter	
Linear dynamic range/µg L^−1^	20–250
Calibration graph	
Intercept	3.0769
Slope/L µg^–1^	1.8754
Correlation coefficient	0.9997
Detection limit/µg L^−1^	6
Quantification limit/µg L^−1^	20
Repeatability (%)	4.5
Intermediate precision (%)	7.8
Sample throughput (samples h^−1^)	8

**Table 2:** An overview on reported determination of TCP.

Technique	Sample	Sample Treatment	DL	RSD (%)	Ref.
LC–MS/MS	Cucumber, soil	QuEChERS	0.71 µg kg^−1^	<13.2	[4]
LC–MS/MS	Tea	QuEChERS	50 µg kg^−1^ *	≤7.2	[5]
UHPLC–MS/MS	Edible fungi	QuEChERS	0.08 µg kg^−1^	≤4.3	[7]
LC–MS/MS	Lettuce, orange	SLE	10 µg kg^−1^ *	≤19	[11]
UHPLC–MS/MS	Lettuce	QuEChERS	2 µg L^−1^	<6	[12]
SMEKC	Cucumber	DLLME	0.8 µg kg^−1^	≤6.5	[15]
SWV	River water		270 µg L^−1^	<5	[14]
ELISA	Water, soil, pear, tomato	SLE	0.47 µg L^−1^	≤10	[16]
TRFIA	Water, tomato, pear, soil	SLE	0.0019 µg L^−1^	≤11.3	[17]
TSL	Tea	SLE, SPE	60 µg L^−1^	<5	[18]
Fluorescence	Waters		30 µg L^−1^	<5	[19]
PICL	Waters	SPE	0.8 µg L^−1^	<10	[20]
TSL	Waters		60 µg L^−1^	<4	[28]
Proposed	Lettuces	QuEChERS	6 µg L^−1^	≤4	

* Limit of quantification; DL: detection limit; RSD: relative standard deviation; LC–MS/MS: liquid chromatography–tandem mass spectrometry; UHPLC–MS/MS: ultra-high performance liquid chromatography–tandem mass spectrometry; SMEKC: sweeping micellar electrokinetic chromatography; SWV: square-wave voltammetry; ELISA: enzyme-linked immunosorbent assay; TRFIA: time-resolved fluoroimmunoassay; TSL: terbium-sensitized luminescence; PICL: photo-induced chemiluminescence; QuEChERS: quick, easy, cheap, effective, rugged, and safe; SLE: solid–liquid extraction; DLLME: dispersive liquid–liquid microextraction; SPE: solid-phase extraction.

**Table 3:** Interference study carried out for 50 µg L^−1^ TCP.

Foreign Species	Tolerance Interferent/Analyte (*w*/*w*) Ratio
Clothianidin	75
Carbendazim, thiamethoxam	20
o-phenylphenol, cypermethrin, λ-cyhalothrin	6
Acetamiprid, chlorpyrifos, imidacloprid, nitenpyram	1

**Table 4:** Recovery study of TCP in lettuce samples.

Sample	Spiked (mg kg^−1^)	Found (mg kg^−1^)	Recovery ± RSD (%)
	1	0.99 ± 0.02	99 ± 2
Iceberg lettuce-1	2	2.12 ± 0.06	106 ± 3
	4	4.32 ± 0.08	108 ± 2
	0.8	0.83 ± 0.03	104 ± 4
Iceberg lettuce-2	5	5.25 ± 0.1	105 ± 3
	8	7.8 ± 0.3	97 ± 4
	1	0.99 ± 0.03	99 ± 3
Baby Romaine lettuce-1	2	1.82 ± 0.07	91 ± 4
	4	4.1 ± 0.1	102 ± 3
	1	0.93 ± 0.03	93 ± 3
Baby Romaine lettuce-2	4	3.9 ± 0.1	98 ± 4
	6	6.1 ± 0.2	102 ± 3
	0.8	0.74 ± 0.03	93 ± 4
Green oak leaf lettuce-1	3	3.15 ± 0.09	105 ± 3
	6	5.8 ± 0.2	96 ± 3
	1	0.98 ± 0.02	98 ± 2
Green oak leaf lettuce-2	4	3.8 ± 0.1	96 ± 3
	7	7.4 ± 0.3	106 ± 4

## Supplementary Materials

Supplementary File 1
